# Problem-method fit in forest policy analysis: Empirical pre-orientation for selecting tested or innovative social-qualitative methods

**DOI:** 10.1016/j.mex.2020.100794

**Published:** 2020-01-18

**Authors:** Dwi Laraswati, Sari Rahayu, Andita A. Pratama, Emma Soraya, Muhammad A.K. Sahide, Ahmad Maryudi

**Affiliations:** a Faculty of Forestry, Universitas Gadjah Mada, Yogyakarta, Indonesia; b Faculty of Forestry, Universitas Hasanuddin, Makassar, Indonesia

**Keywords:** A rapid selection of research methods for forest policy analysis, Rapid appraisal, Qualitative research, Forest governance, Social research, Method selection, Innovative method

## Abstract

An array of research methods has been employed for social-qualitative inquiries. However, the selection of specific research methods has rarely been given adequate attention. We mapped out the variety of research methods used in social-qualitative inquiries used in the study of forest policy. Our “problem-method fit” map is based on the usage quantity of a method employed in specific forest policy research themes and contextual analyses. Our map provides a suitable basis for rapid appraisal before deciding appropriate research methods for future studies. While the map provides only an indication of the appropriate methods, it may be supplemented and adapted case-by-case according to the specific needs of the research theme.

• We mapped the commonly used research methods in forest policy analysis
• The map is “problem-method fit” for specific policy themes and contextual analyses
• It can be used as for rapid appraisal when choosing appropriate research methods

## Graphical abstract

**Figure:**
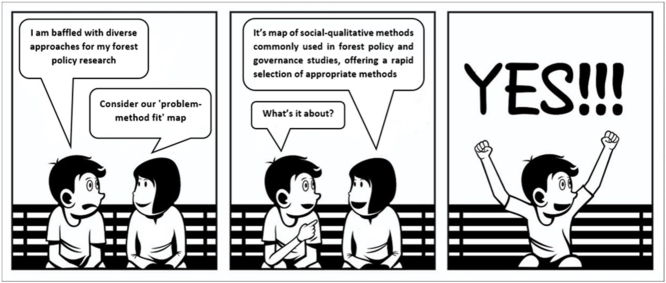

**Specification Table**

**Table:** 

**Subject Area:**	Social Sciences
**More specific subject area:**	*Forest policy and governance*
**Method name:**	*A rapid selection of research methods for forest policy analysis*
**Name and reference of original method:**	*Not applicable*
**Resource availability:**	*Not applicable*

## Method details

Good preparation is one of the key steps in the methodology of any research. Yet, this has not been given sufficient explicit attention [1]. The preparatory stage includes developing study designs and selecting research methods that fit with the focal topic [2,3]. There has been increasing effort to make methodological innovations in social-qualitative inquiries over the past few years. As a result, the repertoire of research methods has become increasingly diverse [3,4]. Researchers now face challenges in selecting methods that are specifically relevant for their studies. Different philosophical paradigms lead to different research methods and designs [[1], [2], [3],^5^]. In this short note, we mapped the social-qualitative methods commonly used in forest policy studies. There is a vibrant social-qualitative research in the field of forest policy [6,7]. Our map is based on the popularity of specific methods used in research themes; as well as the typical analyses used in the field. On the one hand, it identifies tested/ standardized methods commonly used by previous scholars. On the other hand, it can point to methods that are seldom employed to date but potentially useful to find new insights. Either way, this map will help forest policy researchers to better organize and scope their studies.

## Social research methods used in forest policy analysis

### Research problem-method fit

Research is a systematic inquiry to discover new knowledge through describing, explaining, and predicting a certain phenomenon using suitable methods. Hence, it is not the mere gathering of data or information, and then analyzing and interpreting them. Instead, research should be guided by some certain philosophical assumptions, and follows specific procedures to collect relevant data and information [5,8]. The choice of data collection methods depends on the paradigm of the research, and the nature of the problems and questions [2,5,9].

During the data collection phase, researchers make effort to obtain quality data for testing their hypothesis and eventually making conclusions. Certain research methods are relevant when they can be used to gather data that can meaningfully explain the specific problems and provide answers to them. Relevance is often specified as the degree to which a certain method is applicable to the inquiry [10,11]. In this short note, we do not assess the existing research methods based on those attributes*. Instead,* we focus on the extent to which particular methods have been used in specific inquiries. In fact, researchers cannot ignore previous scientific studies on their topics and their employed methods [12]. They may learn from and replicate what has been used by previous researchers.

### Mapping research themes and typical analysis in forest policy

Before organizing the methods in forest policy research, we need to identify typical analyses or inquiries already conducted in the field. Diverse research programs have been emerging in the field of forest policy over the past few years, employing a vast number of social science disciplines such as political science, anthropology, human geography, sociology, environmental history, and legal studies [6]. This has resulted in the use of different study methods. As an illustration, we examine the case of decentralized community forestry policy. This topic has become a prominent topic of forest policy, and has been explored through an array of perspectives using a variety of methods [7].

We analyzed the peer-reviewed journal articles indexed in the Scopus database. We found a total of 1070 scientific articles related to the topic of decentralized community forestry policy, published between 1991 and 2018, 142 of which used purely qualitative approaches^1^ . We thoroughly read all articles, instead of simply skimming the abstracts, for two primary reasons. 1) The abstracts of several articles, particularly those published prior the 2000s, rarely gave mention to the study methods used. 2) We aimed to investigate how data and information were presented and discussed. From the qualitative-based scientific articles, we identified two types of analysis; i.e., core forest policy themes and contextual analysis (see Table 1). The former refers to the main subjects or themes being analyzed, while the latter is defined as the additional analysis required to further support the main arguments.

**Fig. 1:**
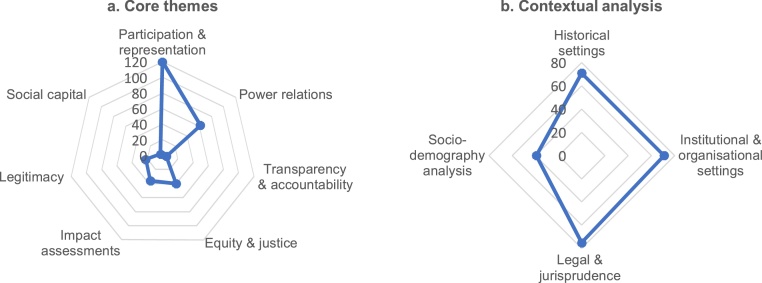
Distribution of core themes and contextual analysis in social-qualitative studies of community forestry policy.

We found 2–3 themes and 3–4 contextual analyses in each article. Participation and representation, and power relations dominate the social-qualitative inquiries in the field of community forestry policy (Fig. 1a). Equity and justice, and impact assessments have also become popular themes in the field to date. In contrast, the themes of legitimacy as well as transparency and accountability remain understudied. They are fundamental issues of good forest governance [13] and can thus be a good value for future research. Similarly, social capital is rarely studied, suggesting a novel and original research area for social-qualitative scientists in the field of community forestry policy. We also found four typical contextual analyses, i.e. historical settings, institutional and organizational setting, legal jurisprudence, and socio-demographic analysis. In contrast to the case of policy and governance themes, the four contextual analyses are equally popular, depending on the focal topics (Fig. 1b).

### The “problem-method fit” map

We delved into the commonly used study methods and accepted by the wider research communities in the field, and mapped them accordingly to the two aforementioned types of analysis. As previously mentioned, the map presented here is based on a review of the extant studies with the goal of identifying usage trends in the study methods. The “problem-method fit” is based on the quantity of usage; i.e., the extent to which a specific method has been employed in a particular forest policy theme. It is important to note that some articles employ multiple methods. In addition, in some papers we found it quite difficult to distinguish whether a method was specifically used for core themes, or contextual analyses, or both. In such case, we consider the methods were used for both. Further, as we seek to find trends on the popular (highly-used) methods, we leave the significantly under-studied (occurrence less than 5) policy themes (social capital, transparency & accountability) out of the analysis to avoid biases.

**Fig. 2:**
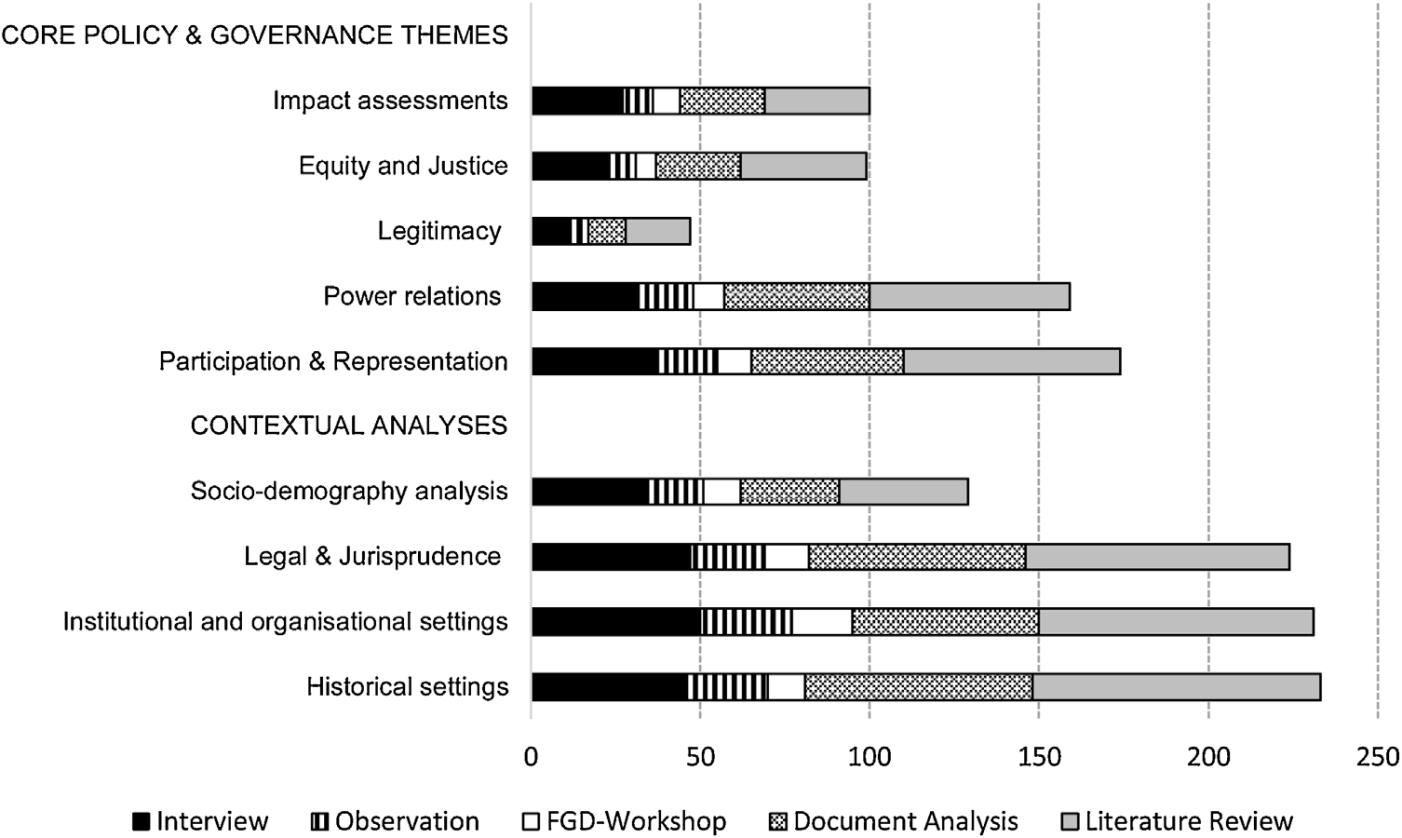
Uses of methods in social-qualitative studies of community forestry policy.

Fig. 2 shows that interview, document analysis and review of literature are among the overriding methods as they were frequently used in both the core policy themes and contextual analyses. In contrast, focused group discussions and workshops were relatively underused in the research field. Fig. 2 also suggests that specific methods seem to be particularly conducive to certain types of inquiries. Document (secondary data) analysis and review of literature are more common for the contextual analyses, while observations seems a good fit for core policy themes.

Thus, understanding the nature of the specific themes being analyzed is the key to researchers of forest policy in order to make judgments on the types of data and the collection methods required. Our findings can be useful in revealing specific study methods applicable to specific themes and analyses. It can be very useful for future qualitative research in forest policy. It provides an applicable basis for a rapid appraisal (pre-orientation) before decision making on tested research methods and strategies.

## Responsible role of researchers in method selection and adaptation

Instead of being an easy-fix for any research problem, our findings provide guidance and indicates trends of popular methods only. The decision on the method needs to be critically checked and possibly supplemented with additional methods by the researcher. Researchers may follow the trends found in this paper and use the most frequently used (i.e. popular) methods. It is fairly suitable for different and highly new empirical cases. Nonetheless, quality research is often measured by its novelty and originality, in terms of new methods, data, or new understanding about specific social phenomenon. In fact, similar research is done too often, providing limited novel and original ideas.

There is a possibility of new methodological innovations as the nature of research in forest policy continues evolving [14]. Trying a new set of methods that is rarely used to date can be crucial for researchers to find new insights. We thus encourage researchers to critically and realistically consider method innovation or adaptation. For instance, as we found FGD and workshop are limitedly used to date, but they are appropriate to gain data from purposely selected individuals [15]. They can be more used in the future, for instance for the theme of power relations, and potentially justice issues, since power analysis is more fruitful when dealing with few powerful actors in specific policy networks [2]. Document analysis and literature review, which we found are more used in contextual analysis, can potentially be used for core policy themes. Their values have been analyzed [7], and there is an emerging forest policy research employing the methods [see 16,17,18].

## Conclusion

Given the potential challenges facing researchers in forest policy due to the diverse research methods available, we mapped out these methods as a basis that is specifically tailored for forest policy analysis. This map was developed based on the popular use of specific approaches in specific forest policy research themes, generated from the rich body of literature in the field. Our map can be used as a rapid appraisal guide for researchers to develop specific strategies for their studies more efficiently. However, researchers need to critically assess its suitability for their studies. They are also encouraged to explore possibilities to use the less standardized methods as new innovations in the research.

## Declaration of Competing Interest

All authors have participated in the conception and design and writing of the article, and have no affiliation with any organization with a direct or indirect financial interest in the subject matter discussed in the manuscript.

## Footnotes

1 The following keywords were used in the search: (“community forestry” OR” social forestry” OR “Community forest tenure” OR” small scale forestry” OR “smallholder forestry” OR” community based forestry” OR” community based forest management” OR “customary forest” OR “village forest” OR “rural forest”) AND* Policy
